# Association between different insulin resistance surrogates and all-cause mortality in patients with coronary heart disease and hypertension: NHANES longitudinal cohort study

**DOI:** 10.1186/s12933-024-02173-7

**Published:** 2024-02-28

**Authors:** Xin-Zheng Hou, Yan-Fei Lv, Yu-Shan Li, Qian Wu, Qian-Yu Lv, Ying-Tian Yang, Lan-Lan Li, Xue-Jiao Ye, Chen-Yan Yang, Man-Shi Wang, Lin-Lin Cao, Shi-Han Wang

**Affiliations:** 1 Department of Cardiovascular Diseases, Guang ‘anmen Hospital, China Academy of Chinese Medical Sciences, Beijing, China; 2 College of Management, Fudan University, Shanghai, China; 3 College of Chinese Medicine, Beijing University of Chinese Medicine, Beijing, China; 4 Department of Cardiovascular Diseases, Guangwai Hospital, Beijing, China

**Keywords:** Coronary heart disease, Hypertension, Insulin resistance, All-cause mortality

## Abstract

**Background:**

Studies on the relationship between insulin resistance (IR) surrogates and long-term all-cause mortality in patients with coronary heart disease (CHD) and hypertension are lacking. This study aimed to explore the relationship between different IR surrogates and all-cause mortality and identify valuable predictors of survival status in this population.

**Methods:**

The data came from the National Health and Nutrition Examination Survey (NHANES 2001–2018) and National Death Index (NDI). Multivariate Cox regression and restricted cubic splines (RCS) were performed to evaluate the relationship between homeostatic model assessment of IR (HOMA-IR), triglyceride glucose index (TyG index), triglyceride glucose-body mass index (TyG-BMI index) and all-cause mortality. The recursive algorithm was conducted to calculate inflection points when segmenting effects were found. Then, segmented Kaplan–Meier analysis, LogRank tests, and multivariable Cox regression were carried out. Receiver operating characteristic (ROC) and calibration curves were drawn to evaluate the differentiation and accuracy of IR surrogates in predicting the all-cause mortality. Stratified analysis and interaction tests were conducted according to age, gender, diabetes, cancer, hypoglycemic and lipid-lowering drug use.

**Results:**

1126 participants were included in the study. During the median follow-up of 76 months, 455 participants died. RCS showed that HOMA-IR had a segmented effect on all-cause mortality. 3.59 was a statistically significant inflection point. When the HOMA-IR was less than 3.59, it was negatively associated with all-cause mortality [HR = 0.87,95%CI (0.78, 0.97)]. Conversely, when the HOMA-IR was greater than 3.59, it was positively associated with all-cause mortality [HR = 1.03,95%CI (1.00, 1.05)]. ROC and calibration curves indicated that HOMA-IR was a reliable predictor of survival status (area under curve = 0,812). No interactions between HOMA-IR and stratified variables were found.

**Conclusion:**

The relationship between HOMA-IR and all-cause mortality was U-shaped in patients with CHD and hypertension. HOMA-IR was a reliable predictor of all-cause mortality in this population.

## Background

Coronary heart disease (CHD) is a significant challenge facing global public health. According to the American Heart Association, more than 350,000 people die of CHD in the United States each year [1]. Hypertension is the leading cause of major cardiovascular adverse events [2]. For patients with both CHD and hypertension, early identification and intervention of risk factors that affect prognosis are crucial for reducing the global burden of cardiovascular diseases [3].

Insulin resistance (IR) is a prominent feature of metabolic syndrome, referring to a decrease in the efficiency of insulin in promoting glucose utilization [4]. IR is considered a risk factor for microvascular and macrovascular lesions [5]. The hyperinsulinemic-normal glucose clamp test is the gold standard for IR measurement, but it is a complex and invasive examination that is not suitable for clinical research [6]. A validated alternative evaluation index is the homeostatic model assessment of insulin resistance (HOMA-IR), which is calculated from fasting blood glucose and insulin concentrations [7]. However, circulating insulin concentrations are not routinely measured in primary care, so various simple and feasible alternative evaluation indices for IR have emerged, such as the triglyceride glucose index (TyG index), triglyceride glucose-body mass index (TyG-BMI index) [8, 9]. Some studies have found that the TyG index is associated with in-hospital all-cause mortality in patients with severe CHD [10]. However, few studies have evaluated the correlation between these IR surrogates and all-cause mortality in patients with CHD and hypertension. Only a few related studies conducted in China have found that the TyG index is associated with short-term adverse CVD outcomes in patients with CHD and hypertension [11]. There is still a lack of evidence on which IR surrogates can serve as long-term predictors of all-cause mortality risk in patients with CHD and hypertension.

This study linked the National Health and Nutrition Examination Survey (NHANES) and National Death Index (NDI) data to investigate the relationship between different IR surrogates and long-term all-cause mortality in patients with CHD and hypertension. The aim is to identify valuable predictors of survival status in this population.

## Methods

### Study design

The baseline data was obtained from NHANES. NHANES is a continuous cross-sectional survey with national representation and complex multi-stage sampling, aiming to assess the nutritional and health status of the non-institutionalized US population. NHANES contains a large amount of data on demographics, dietary nutrition, physical examination, laboratory examination, and medical history. Detailed information about NHANES has been described in other studies [12]. We collected the information of participants who were first interviewed between 2001 and 2018. Then we linked the National Death Index (NDI) of the National Center for Health Statistics (NCHS) to obtain the survival status of the participants, and constructed a NHANES longitudinal follow-up cohort. The NCHS Ethical Review Board approved the study. Informed consent was obtained from all study participants [13]. Therefore, no additional informed consent and ethical review were required for our research.

### Study population

Participants who were surveyed between 2001 and 2018 and had both CHD and hypertension were included in this study. Those missing IR surrogates and survival status were excluded. The history of CHD and hypertension was obtained through interviews. CHD information was obtained by asking participants:”Has a doctor or other health professional ever told you that you had coronary heart disease?”” Has a doctor or other health professional ever told you that you had angina, also called angina pectoris?” or “Has a doctor or other health professional ever told you that you had a heart attack (also called myocardial infarction?” If they answered "Yes" to any of the above questions, they were diagnosed CHD. Similarly, the information on hypertension was obtained through self-report of having been diagnosed with hypertension by a doctor or currently taking antihypertensive prescription drugs. Participants who had both CHD and hypertension were included in this study.

### IR surrogates

The IR surrogates in this study include the HOMA-IR, TyG index, and TyG-BMI index. The HOMA-IR was calculated as follows: HOMA-IR = fasting glucose (mmol/L) × fasting insulin (µU/mL)/22.5 [14]. The TyG index was calculated as follows: TyG = Ln [fasting triglycerides (mg/dL) × fasting glucose (mg/dL)/2] [15]. The TyG-BMI index was calculated as follows: TyG-BMI = TyG index × BMI (kg/m2) [16]. Trained laboratory personnel collected fasting blood from the participants. The blood samples were frozen at -20 °C and transported to the laboratory for testing. Fasting insulin was measured using the AIA-PACK IRI. The AIA-PACK IRI is a two-site immunoenzymometric assay, which is performed on Tosoh AIA System analyzer. The measurement of triglycerides and fasting glucose were measured through enzymatic assays on Roche Modular P and Roche Cobas 6000 chemistry analyzers, respectively. BMI was calculated using a formula that takes into account the participant's standing height and weight.

### Survival status

Using a series of identifiers such as social security number and date of birth, NCHS used probabilistic matching to link NHANES with NDI data to obtain survival status data for participants. The follow-up of participants was terminated on December 31, 2019. If there was no match with the NDI, it was assumed that the person was alive [17]. This study considered both survival outcomes and survival time.

### Covariates

Demographic, medical history, and laboratory blood test data of participants were collected. Demographic data included age, gender, race, education level, marital status, and income -poverty ratio (PIR). Medical history information included diabetes, cancer, heart failure, stroke, chronic obstructive pulmonary disease (COPD), use of hypoglycemic and lipid-lowering prescription drugs, tobacco use, BMI, waist circumference, and hip circumference. Laboratory blood test data included low density lipoprotein cholesterol (LDL-C), high density lipoprotein cholesterol (HDL-C), total cholesterol (TC), alanine aminotransferase (ALT), albumin, alkaline phosphatase (ALP), aspartate aminotransferase (AST), urea nitrogen, creatine kinase (CK), creatinine (Cr), gamma-glutamyltransferase (GGT), lactate dehydrogenase (LDH), iron, phosphorus, potassium, sodium, calcium, total bilirubin, uric acid, glycosylated hemoglobin (HbA1c), hemoglobin (Hb), platelet count, and white blood cell count (WBC). The demographic and medical history information was obtained through interviews. Smoking more than 100 cigarettes in a lifetime was defined as a tobacco user. BMI, waist circumference, and hip circumference were obtained through measurement. The definition of diabetes was self-reported diagnosis, use of insulin or oral hypoglycemic agents, fasting glucose ≥ 7 mmol/L, or HbA1c ≥ 6.5% [18].

### Statistic analysis

Participants were divided into two groups according to survival status to describe the characteristics of the study population. Continuous variables were expressed as mean and standard deviation or median and quartile, and the t-test or Kruskal–Wallis rank sum test was selected for hypothesis testing according to applicable conditions. Classified variables were expressed as absolute numbers and percentages, and the chi-square test was used for hypothesis testing.

Multivariable Cox regression models were used to evaluate of the linear relationship between different IR surrogates and survival status. We constructed three regression models by adjusting different covariates to control for confounding biases. The selection of covariates was driven both theoretically and statistically. Some covariates theoretically associated with survival status were fixed in the model, such as age, gender, race, diabetes, and cancer. Other variables were selected using statistical methods. First, variables with variance inflation factors greater than 5 were excluded to avoid multicollinearity. Then, a two-way effect change method was used to screen variables that had an impact on the effect size of the independent variables greater than 10%. Adjust I adjusted for age, gender, race, diabetes, and cancer, and Adjust II was a fully adjusted model. Multiple chain interpolation was used to fill in missing data.

Multivariable restricted cubic splines (RCS) were used to identify the nonlinear relationship between different IR surrogates and survival status. We aimed to identify potentially valuable predictors of survival status based on the shape of the RCS curves. Recursive algorithms were used to calculate potential cut-off points. Based on these cut-off points, we conducted segmented Kaplan–Meier analysis, LogRank tests, and multivariable Cox regression.

In addition, we evaluated the discrimination ability and accuracy of the fully adjusted model using receiver operating characteristic (ROC) curves, area under the curve (AUC), and calibration curves. We compared the differences in AUC using the Z-test. Finally, we conducted stratified analyses and interaction tests based on age, gender, diabetes, cancer, and the use of hypoglycemic and lipid-lowering prescription drugs.

Data analysis was completed by software IBM SPSS Statistics,(version 26.0) and R software (version 4.2.1). P < 0.05 on both sides was considered statistically significant. Taking into account the complex sampling design of NHANES, the minimum subsample weights, clustering, and stratification were included in the analysis [19].

## Results

### General characteristics of participants

**Fig. 1:**
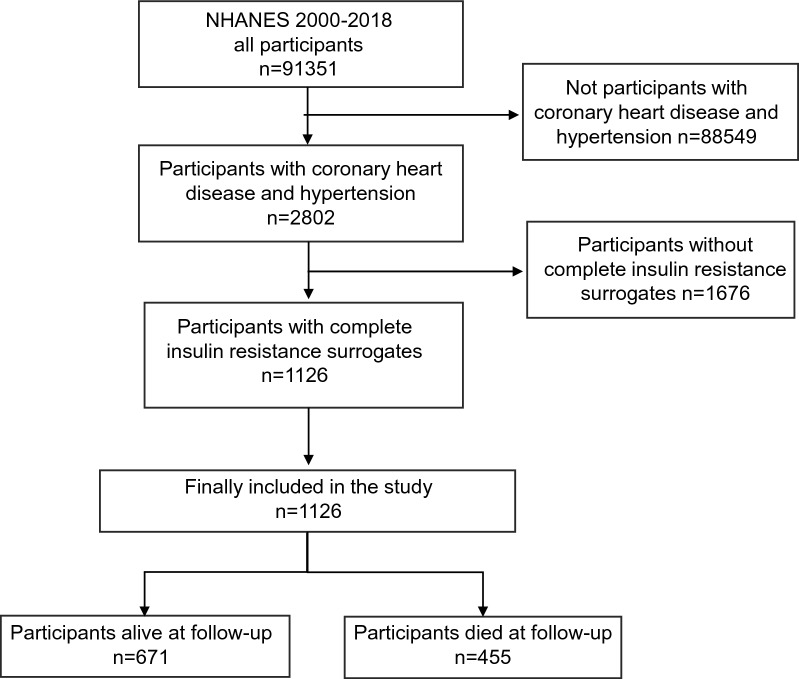
Study population screening flow chart

The screening process for the study population is presented in Fig. 1. 1126 participants were included in the analysis. 1126 participants provided follow-up data for a total of 96,048 person-months. And during the median follow-up of 76 months, 455 participants died. They had lower HOMA-IR and TyG-BMI index at baseline, but the difference in TyG index was not statistically significant. In addition, they were older, had a higher proportion of males, and had higher prevalences of diabetes and cancer. Detailed information on the demographics, medical history, laboratory tests at baseline, and the results of univariate analysis, are presented in Tables 1 and 2.

**Table 1:** Demographic and medical history baseline characteristics

Variables	Surviving participants (n = 671)	Dead participants (n = 455)	P-value
Age.years	64.36 (11.38)	72.48 (9.63)	< 0.001
Gender			0.031
Male	380 (56.63%)	287 (63.08%)	
Female	291 (43.37%)	168 (36.92%)	
Race			< 0.001
Mexican American	75 (11.18%)	31 (6.81%)	
Other Hispanic	66 (9.84%)	21 (4.62%)	
Non-Hispanic White	331 (49.33%)	308 (67.69%)	
Non-Hispanic Black	145 (21.61%)	78 (17.14%)	
Other Race	54 (8.05%)	17 (3.74%)	
Marriage			< 0.001
Having a partner	416 (62.00%)	228 (50.11%)	
Without partner	255 (38.00%)	227 (49.89%)	
Education			0.034
High school and below	224 (33.38%)	180 (39.56%)	
Above high school	447 (66.62%)	275 (60.44%)	
PIR	2.32 (1.58)	2.13 (1.41)	0.047
BMI	30.71 (6.39)	29.19 (6.49)	< 0.001
Hip circumference.cm	34.16 (4.90)	32.56 (5.28)	< 0.001
Waist circumference.cm	106.32 (14.67)	104.56 (15.65)	0.062
Diabetics			< 0.001
Yes	609 (90.76%)	441 (96.92%)	
No	62 (9.24%)	14 (3.08%)	
Cancer patients			< 0.001
Yes	119 (17.73%)	126 (27.69%)	
No	552 (82.27%)	329 (72.31%)	
Heart failure patients			< 0.001
Yes	163 (24.62%)	160 (35.71%)	
No	499 (75.38%)	288 (64.29%)	
Stroke patients			0.016
Yes	104 (15.50%)	96 (21.10%)	
No	567 (84.50%)	359 (78.90%)	
COPD patients			0.481
Yes	58 (8.64%)	34 (7.47%)	
No	613 (91.36%)	421 (92.53%)	
Hypoglycemic drugs or insulin Users			0.012
Yes	548 (81.67%)	397 (87.25%)	
No	123 (18.33%)	58 (12.75%)	
Lipid-lowering drug users			0.574
Yes	624 (93.00%)	427 (93.85%)	
No	47 (7.00%)	28 (6.15%)	
Tobacco users			0.023
Yes	399 (59.46%)	301 (66.15%)	
No	272 (40.54%)	154 (33.85%)	

Mean(SD) | Median (Q1-Q3) | N(%). COPD: chronic obstructive pulmonary disease, PIR: income-poverty ratio, BMI: body mass index

**Table 2:** Baseline characteristics of laboratory blood test

Variables	Surviving participants (n = 671)	Dead participants (n = 455)	P-value
LDL-C.mmol/L	2.61 (0.97)	2.50 (0.95)	0.047
HDL-C.mmol/L	1.29 (0.39)	1.34 (0.44)	0.081
TG.mg/dL	123.00 (87.50–179.50)	119.00 (84.00–169.00)	0.261
TC.mmol/L	4.45 (3.80–5.23)	4.37 (3.71–5.20)	0.393
Fasting blood glucose.mmol/L	6.49 (2.51)	6.53 (2.17)	0.795
Albumen.g/L	41.36 (3.20)	40.63 (3.77)	< 0.001
ALP. IU/L	72.00 (59.00–87.14)	74.38 (62.36–89.00)	0.011
AST.U/L	23.00 (19.00–28.00)	23.00 (19.50–28.00)	0.318
ALT.U/L	21.00 (16.00–28.00)	19.00 (15.00–25.00)	0.048
CK.IU/L	126.00 (85.54–178.90)	128.34 (94.15–166.85)	0.049
Creatinine.umol/L	84.86 (69.84–102.54)	97.24 (79.56–123.76)	< 0.001
GGT.IU/L	23.00 (17.00–34.00)	22.00 (16.00–37.00)	0.061
LDH.IU/L	138.00 (122.00–156.00)	139.00 (125.50–161.00)	0.449
Serum urea nitrogen.mmol/L	5.36 (4.28–7.14)	6.43 (4.64–8.93)	< 0.001
Total bilirubin.umol/L	10.26 (8.55–13.68)	11.97 (10.26–15.39)	< 0.001
Serum uric acid.umol/L	354.84 (87.80)	384.08 (110.50)	< 0.001
Iron.umol/L	14.30 (11.10–18.10)	13.43 (10.20–17.70)	0.074
Calcium.mmol/L	2.35 (0.09)	2.35 (0.11)	0.359
Phosphorus.mmol/L	1.18 (0.18)	1.21 (0.21)	0.008
Potassium.mmol/L	4.08 (0.39)	4.17 (0.43)	0.001
Sodium.mmol/L	139.79 (2.61)	138.86 (2.89)	< 0.001
Hemoglobin.g/dL	14.09 (1.51)	13.78 (1.79)	0.002
Platelet count.1000 Cells /uL	216.00 (182.00–261.50)	216.00 (182.00–263.50)	0.939
White blood cell count.1000 Cells /uL	6.70 (5.70–8.10)	7.20 (5.70–8.80)	0.006
Fasting insulin.μU/mL	12.44 (7.85–20.05)	10.36 (6.73–18.30)	0.055
HbA1c.%	6.25 (1.31)	6.28 (1.34)	0.631
HOMA-IR	3.47 (1.99–5.69)	2.82 (1.65–5.24)	0.446
TyG	8.82 (8.39–9.26)	8.80 (8.37–9.22)	0.477
TyG-BMI	266.16 (226.28–310.56)	251.23 (214.75–294.37)	< 0.001

Mean(SD) | Median (Q1-Q3)

*LDL-C* low density lipoprotein cholesterol, *HDL-C* high density lipoprotein cholesterol, *TG* triglycerides, *TC* total cholesterol, *ALP* alkaline phosphatase, *AST* aspartate aminotransferase, *ALT* alanine aminotransferase, *CK* creatine kinase, *GGT* gamma-glutamyltransferase, *LDH* lactate dehydrogenase, *HbA1c* glycosylated hemoglobin, *HOMA-IR* homeostatic model assessment of insulin resistance, *TyG* triglyceride glucose index, *TyG-BMI* triglyceride glucose- body mass index

### Association between different IR surrogates and survival status

**Fig. 2:**
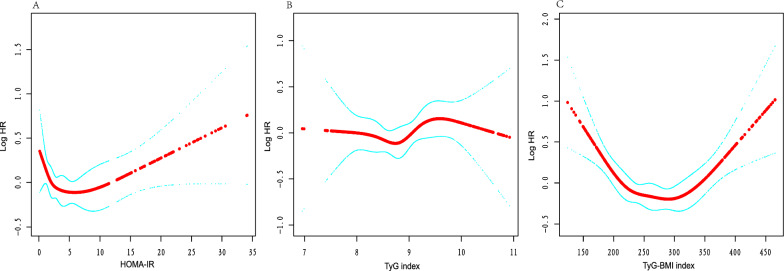
Nonlinear relationship between different IR surrogates and all-cause mortality. The red line in the figure represents Log(HR), and the blue line represents the 95% confidence interva. **A** HOMA-IR, **B** TyG index, and **C** TyG-BMI index

The following variables were adjusted in Adjust II model: age, gender, race, diabetes, cancer, marriage, education, LDL-C, HDL-C, albumen, ALP, AST, CK, creatinine, GGT, LDH, iron, phosphorus, sodium, potassium, calcium, total bilirubin, uric acid, urea nitrogen, hemoglobin, platelet count, white blood cell count, stroke, COPD, hypoglycemic drugs or insulin use, lipid-lowering drug use. The results of the multivariable Cox regression and RCS are presented in Table 3 and Fig. 2, respectively. In the fully adjusted Cox regression model, when IR surrogates were included as continuous variables, their relationship with survival status was not statistically significant. The RCS curve demonstrated the nonlinear relationship between IR surrogates and survival status. Figure 2A showed that the HOMA-IR may have a piecewise effect on survival status with a distinct inflection point, indicating that it may be a predictor of survival status.

**Table 3:** Relationship between different IR surrogates and all-cause mortality

Exposure	Non-adjusted	Adjust I	Adjust II
HOMA-IR	0.99 (0.97, 1.01) 0.1757	1.01 (0.98, 1.03) 0.6197	1.01 (0.99, 1.03) 0.3484
TyG	0.92 (0.79, 1.06) 0.2444	0.99 (0.84, 1.16) 0.8762	1.05 (0.88, 1.25) 0.5931
TyG-BMI	1.00 (1.00, 1.00) < 0.0001	1.00 (1.00, 1.00) 0.2280	1.00 (1.00, 1.00) 0.2329

hazard ratio(HR), 95% confidence interval(CI),and P-value. Adjust I: Age, sex, race, diabetes, and cancer. Adjust II: Age, sex, race, diabetes, cancer, and variables obtained by using the two-way effect change method

### The relationship between HOMA-IR and survival status

**Fig. 3:**
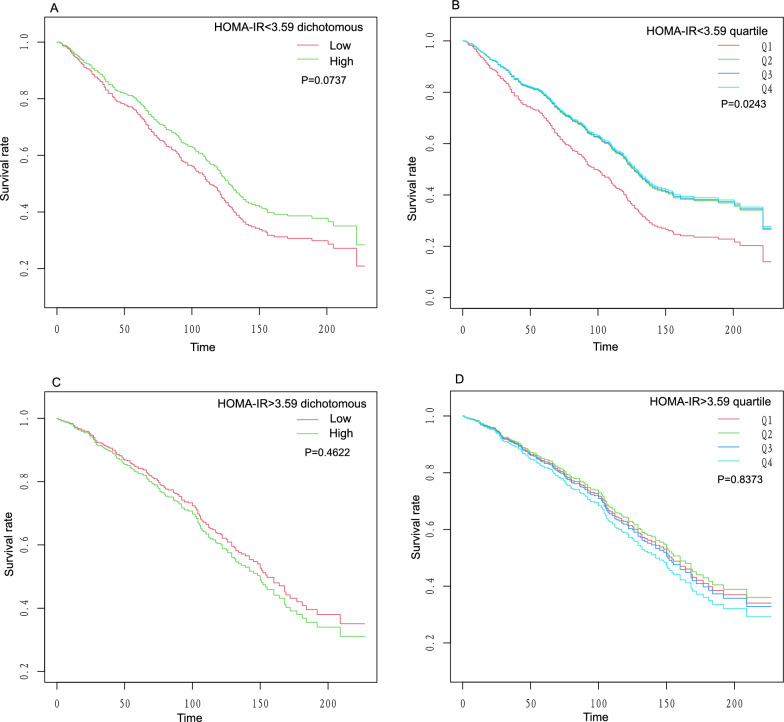
Segmented HOMA-IR survival curve. **A** HOMA-IR < 3.59 dichotomous, **B** HOMA-IR < 3.59 quartile, **C** HOMA-IR > 3.59 dichotomous, and **D** HOMA-IR > 3.59 quartile

Using recursive partitioning analysis, we found a statistically significant breakpoint (breakpoint = 3.59, P = 0.005) in the relationship between the HOMA-IR and survival status. When the HOMA-IR is less than 3.59, it was negatively associated with survival status [HR = 0.87,95%CI (0.78, 0.97)]. Conversely, when the HOMA-IR was greater than 3.59, it was positively associated with survival status [HR = 1.03,95%CI (1.00, 1.05)]. These results are presented in Table 4. Taking 3.59 as the cut-off point, we further studied the relationship between HOMA-IR and survival status in segments. The results of the segmented Kaplan–Meier analysis are shown in Fig. 3. When the HOMA-IR was less than 3.59, both binary and quartile classification of HOMA-IR were associated with low survival rates in individuals with low levels of HOMA-IR (Fig. 3A, B). However, when the HOMA-IR was greater than 3.59, individuals with high levels of HOMA-IR were associated with low survival rates (Fig. 3C, D). In addition, we also convert HOMA-IR into categorical variables for piecewise multivariate Cox regression. The results showed that when the HOMA-IR is less than 3.59, in the Adjust I, the higher HOMA-IR was associated with a lower all-cause mortality rate [HR = 0.69,95%CI (0.54, 0.87)], and the trend test was statistically significant (P = 0.0004). When the HOMA-IR was greater than 3.59, in the Adjust I, the higher HOMA-IR was associated with an increased all-cause mortality rate [HR = 1.44,95%CI (1.07, 1.95)]. These results are presented in Table 5.

**Table 4:** Cut point and segmentation effects of HOMA-IR

Items	Outcome:
Linear effect	1.01 (0.99, 1.03) 0.3484
Segmentation effect	
Cut point (K)	3.59
< K segment effect	0.87 (0.78, 0.97) 0.0091
> K segment effect	1.03 (1.00, 1.05) 0.0177
Effect difference	1.19 (1.06, 1.34) 0.0044
Logarithmic likelihood ratio test	0.005
95%CI of the Cut point	2.86, 4.34

hazard ratio(HR), 95% confidence interval(CI),and P-value

**Table 5:** Segmented Cox regression analysis and trend test of HOMA-IR

	Non-adjusted	Adjust I	Adjust II
HOMA-IR < 3.59			
HOMA-IR dichotomous			
Low	Reference	Reference	Reference
High	0.81 (0.63, 1.02) 0.0751	0.69 (0.54, 0.87) 0.0023	0.96 (0.73, 1.26) 0.7673
HOMA-IR quartile			
Q1	Reference	Reference	Reference
Q2	0.68 (0.49, 0.94) 0.0193	0.69 (0.49, 0.96) 0.0275	0.68 (0.47, 0.99) 0.0460
Q3	0.67 (0.48, 0.93) 0.0159	0.59 (0.43, 0.83) 0.0020	0.82 (0.56, 1.20) 0.3153
Q4	0.65 (0.47, 0.91) 0.0127	0.54 (0.39, 0.76) 0.0004	0.73 (0.49, 1.07) 0.1042
P trend	0.0185	0.0004	0.2615
HOMA-IR > 3.59			
HOMA-IR dichotomous			
Low	Reference	Reference	Reference
High	1.11 (0.83, 1.49) 0.4635	1.44 (1.07, 1.95) 0.0166	1.16 (0.84, 1.60) 0.3774
HOMA-IR quartile			
Q1	Reference	Reference	Reference
Q2	0.95 (0.62, 1.44) 0.8005	0.93 (0.61, 1.43) 0.7427	0.93 (0.59, 1.45) 0.7436
Q3	1.03 (0.68, 1.56) 0.8769	1.37 (0.90, 2.10) 0.1411	1.00 (0.63, 1.60) 0.9870
Q4	1.14 (0.75, 1.73) 0.5312	1.41 (0.92, 2.15) 0.1122	1.22 (0.78, 1.91) 0.3784
P trend	0.3996	0.0618	0.6046

hazard ratio(HR), 95% confidence interval(CI),and P-value. Adjust I: Age, sex, race, diabetes, and cancer. Adjust II: Age, sex, race, diabetes, cancer, and variables obtained by using the two-way effect change method

### Subgroup analysis and model evaluation

**Fig. 4:**
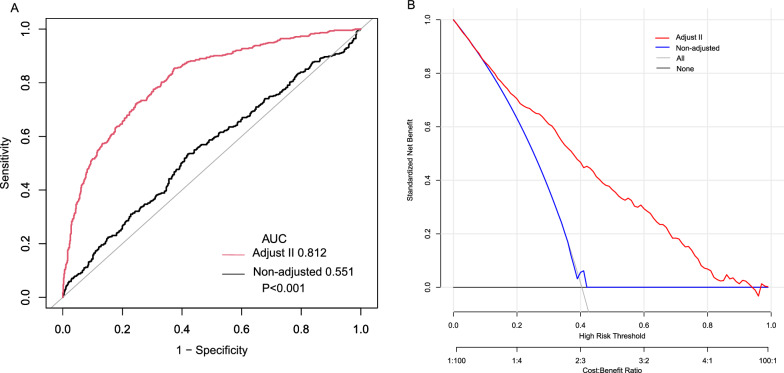
Discrimination and accuracy of HOMA-IR in evaluating all-cause mortality in the fully adjusted model.** A** ROC and AUC,** B** calibration curve

Table 6 presents the results of the segmented subgroup analysis and interaction tests between the HOMA-IR and survival status. Age, gender, diabetes, cancer, hypoglycemic and lipid-lowering prescription drugs did not have significant interactions with the HOMA-IR. The ROC curve and calibration curve in Fig. 4 indicated that when using the HOMA-IR to evaluate survival status, the fully adjusted model we constructed had better discriminatory and accuracy compared to the univariate Cox regression model. The AUC value for the fully adjusted model was 0.812, which was significantly higher than unadjusted model and the difference was statistically significant (P < 0.001).

**Table 6:** HOMA-IR segmented subgroup analysis

	Non-adjusted	Adjust I	Adjust II	P for interaction
HOMA-IR < 3.59				
Age dichotomous				0.4039
Low	0.73 (0.55, 0.99) 0.0398	0.73 (0.54, 0.98) 0.0339	0.81 (0.59, 1.10) 0.1727	
High	0.75 (0.63, 0.89) 0.0012	0.74 (0.62, 0.88) 0.0008	0.94 (0.77, 1.14) 0.5309	
Gender				0.7908
Male	0.79 (0.66, 0.93) 0.0055	0.72 (0.61, 0.86) 0.0002	0.88 (0.72, 1.06) 0.1758	
Female	0.91 (0.69, 1.19) 0.4804	0.87 (0.66, 1.13) 0.2932	0.92 (0.68, 1.24) 0.5745	
Diabetics				0.6544
Yes	0.82 (0.71, 0.95) 0.0089	0.76 (0.65, 0.88) 0.0002	0.88 (0.74, 1.04) 0.1424	
No	1.10 (0.46, 2.59) 0.8329	0.95 (0.37, 2.48) 0.9211	1.09 (0.45, 2.65) 0.8545	
Cancer				0.297
Yes	0.65 (0.49, 0.86) 0.0027	0.63 (0.47, 0.85) 0.0024	0.77 (0.55, 1.06) 0.1065	
No	0.87 (0.73, 1.04) 0.1230	0.81 (0.68, 0.95) 0.0115	0.93 (0.77, 1.12) 0.4529	
Hypoglycemic drugs or insulin Users				0.8085
Yes	0.86 (0.73, 1.01) 0.0583	0.73 (0.62, 0.86) 0.0002	0.88 (0.73, 1.05) 0.1642	
No	0.73 (0.50, 1.07) 0.1105	0.81 (0.56, 1.18) 0.2788	0.93 (0.63, 1.37) 0.7047	
Lipid-lowering drug users				0.96
Yes	0.83 (0.72, 0.97) 0.0162	0.76 (0.66, 0.88) 0.0004	0.89 (0.75, 1.05) 0.1637	
No	0.85 (0.41, 1.77) 0.6620	0.77 (0.36, 1.65) 0.5061	0.90 (0.42, 1.95) 0.7988	
HOMA-IR > 3.59				
Age dichotomous				0.2771
Low	1.01 (0.97, 1.05) 0.5769	1.01 (0.98, 1.05) 0.4820	1.00 (0.96, 1.05) 0.8460	
High	1.03 (1.00, 1.06) 0.0873	1.04 (1.01, 1.07) 0.0094	1.03 (1.00, 1.07) 0.0657	
Gender				0.05
Male	0.99 (0.96, 1.03) 0.7300	1.02 (0.99, 1.06) 0.2589	1.00 (0.97, 1.04) 0.9058	
Female	1.05 (1.01, 1.08) 0.0053	1.05 (1.02, 1.09) 0.0038	1.06 (1.02, 1.10) 0.0040	
Diabetics				0.7656
Yes	1.02 (0.99, 1.04) 0.1842	1.03 (1.01, 1.06) 0.0053	1.03 (1.00, 1.05) 0.0565	
No	0.99 (0.67, 1.44) 0.9381	1.01 (0.67, 1.54) 0.9530	1.10 (0.70, 1.73) 0.6737	
Cancer				0.0349
Yes	1.09 (1.04, 1.15) 0.0010	1.12 (1.05, 1.18) 0.0002	1.10 (1.03, 1.17) 0.0043	
No	1.01 (0.98, 1.04) 0.5390	1.02 (0.99, 1.05) 0.1266	1.01 (0.98, 1.04) 0.3709	
Hypoglycemic drugs or insulin Users				0.0716
Yes	1.01 (0.99, 1.04) 0.2559	1.03 (1.01, 1.06) 0.0109	1.02 (1.00, 1.05) 0.0977	
No	1.09 (0.96, 1.24) 0.1717	1.17 (1.03, 1.32) 0.0131	1.17 (1.03, 1.33) 0.0189	
Lipid-lowering drug users				0.3338
Yes	1.01 (0.99, 1.04) 0.2313	1.03 (1.01, 1.06) 0.0095	1.02 (1.00, 1.05) 0.0741	
No	1.09 (0.96, 1.23) 0.1837	1.11 (0.98, 1.27) 0.1089	1.12 (0.95, 1.32) 0.1699	

hazard ratio(HR), 95% confidence interval(CI),and P-value. Adjust I: Age, sex, race, diabetes, and cancer. Adjust II: Age, sex, race, diabetes, cancer, and variables obtained by using the two-way effect change method. Variables used as stratification criteria were not adjusted

## Discussion

This study explored the relationship between different IR surrogates and all-cause mortality in a community-based population with CHD and hypertension in the United States. Using multivariate Cox regression and RCS analysis, we found that HOMA-IR was a reliable predictor of all-cause mortality risk in patients with CHD and hypertension. HOMA-IR was associated with all-cause mortality risk in a U-shaped manner, and high or low HOMA-IR increased the risk of all-cause mortality in this population.

Baseline characteristics of the study population indicated that the control of blood sugar, blood lipids and weight in patients with CHD and hypertension did not reach optimal standards. Participants who died during follow-up were more likely to be male, older, with lower education level and income, and more smokers, diabetes, heart failure, stroke, and cancer patients. In segmented multivariate Cox regression analysis, when HOMA-IR was modeled as a continuous variable, the effect values were statistically significant. When HOMA-IR was modeled as categorical variables, the effect values were not statistically significant, which may due to a decrease in testing efficiency resulting from insufficient sample size for each group.

IR is an important feature of the metabolic syndrome, and in addition to diabetes, obesity, and dyslipidemia, it is also a risk factor for the development of CVD, and may be associated with adverse outcomes in patients with CVD [20]. Some studies have found that IR is positively associated with the development of CVD in patients with prediabetes [21]. A Korean cohort study with a median follow-up time of 9.83 years found that IR increased the risk of all-cause mortality, cardiovascular mortality, and adverse cardiovascular events in CVD patients by 87%, 133%, and 267%, respectively [22]. The hyperinsulinemic-normal glucose clamp technique is the gold standard for the diagnosis of IR, but due to its limitations, it is difficult to use in large-scale clinical studies. Therefore, various different IR surrogates are widely used in clinical research. HOMA-IR is the most widely used surrogate marker, but its calculation requires the measurement of fasting insulin concentration [23]. The TyG index has also received attention due to its simplicity and ease of use. Previous studies have shown that the TyG index has good sensitivity (96.5%) and specificity (85.0%) for the diagnosis of IR compared to the hyperinsulinemic-normal glucose clamp technique [24]. In addition, the TyG-BMI index is also commonly used in clinical research [25].

Previous studies on the relationship between IR surrogates and CVD outcomes have been extensively conducted, but research in community-based populations with CHD and hypertension is lack. Current studies in patients with CHD and hypertension focus on short-term outcomes in hospitalized patients. For example, two cohort studies conducted in China found that in hospitalized patients with CHD and hypertension, the TyG index was positively associated with adverse outcomes, including all-cause mortality, during one-year follow-up [2, 11]. However, our study did not find an association between the TyG index and all-cause mortality, which may be due to differences in study population and follow-up duration. A study has found that in elderly and female patients with CHD who undergo percutaneous coronary intervention, the TyG-BMI index was positively associated with adverse cardiovascular outcomes [26]. However, there is no research on the correlation between the TyG-BMI index and prognosis in patients with CHD and hypertension. Our study did not find a significant correlation between the two. As for HOMA-IR, we found that it was a good predictor of all-cause mortality in patients with CHD and hypertension. In our study, when the HOMA-IR was less than 3.59, it was negatively associated with the risk of death; while when the HOMA-IR was greater than 3.59, it was positively associated with all-cause mortality. The HOMA-IR was U-shapedly associated with all-cause mortality in patients with CHD and hypertension. The results of model evaluation also indicated that the HOMA-IR had good predictive ability for all-cause mortality. Our results are similar to the conclusions of several previous studies.A 20-year follow-up study found that in non-diabetic Finnish men, those with the highest HOMA-IR had a 69% increased risk of CHD mortality [27]. Another study conducted in Chinese with CHD and diabetes found a positive correlation between the HOMA-IR and the risk score for acute coronary events [28]. Other studies have found that when the HOMA-IR is greater than 3.49, CHD patients have an increased risk of plaque progression in the coronary arteries, which is associated with adverse outcomes in coronary heart disease [29]. Above results indicate that the HOMA-IR is associated with the prognosis of CHD. However, this relationship has not been evaluated in patients with CHD and hypertension, and the nonlinear relationship has not been assessed either. Our study fills these gaps.

Hypertension and atherosclerosis processes interact with each other, which can exacerbate the adverse prognosis of CHD [30]. Therefore, CHD patients with comorbid hypertension should receive greater attention. IR is associated with endothelial dysfunction, abnormal lipid metabolism, excessive sympathetic activation, and systemic inflammatory response. These factors are closely related to the development and poor prognosis of CHD and hypertension [7]. Our study found that the HOMA-IR can serve as a predictor of all-cause mortality risk in patients with CHD and hypertension. High and low HOMA-IR indices were associated with an increased risk of all-cause mortality. This suggests that clinicians can use the HOMA-IR to assess the risk of all-cause mortality in patients with CHD and hypertension and take appropriate measures.

The aforementioned studies have indicated an association between IR and its surrogates with the occurrence and prognosis of CVD. However, further quantitative research is warranted in distinct CVD subpopulations to enhance the precision of utilizing IR surrogates in predicting prognosis among CVD patients by clinicians. CHD and hypertension are atherosclerosis-related conditions that frequently coexist. Currently, little studies exam the relationship between IR surrogates and long-term prognosis in individuals with CHD and hypertension. It remains uncertain whether findings from studies focused on short-term outcomes in this population can be extrapolated to predict long-term prognosis. To address the gap, this study was conducted. Our findings revealed a U-shaped association between HOMA-IR and long-term prognosis in patients with CHD and hypertension, exhibiting a segmented effect. This contrasts with the previously reported linear relationship between IR surrogates and short-term prognosis in this population. These results can aid clinicians in more accurately evaluating prognosis using IR surrogates among individuals with CHD and hypertension.

Our research employed cohort study design, an important method for real world study [31]. The cohort study design has perfect external validity, which means that the research conclusions can be more accurately generalized and applied to the real world [31, 32]. However, as an observational study, potential imbalances in the study population may introduce confounding bias [31]. To mitigate this, we collected a comprehensive array of covariates and utilized rigorous statistical techniques, including multivariable Cox regression analysis, to enhance the robustness of our findings. Subsequent researchers can continue to conduct relevant studies to validate their generalizability and applicability in clinical practice.

The strengths and limitations of this study include the following. First, this study fills some of the current research gaps. It evaluates the relationship between different IR surrogates and long-term all-cause mortality in patients with CHD and hypertension from a community-based perspective, and finds that the HOMA-IR can serve as a good prognostic evaluation index. Second, this study simultaneously assesses both linear and nonlinear relationships between variables, identifies segmented effects between variables, and calculates cut-off points. However, there are also some limitations. First, the diagnosis of CHD and hypertension in this study was obtained through self-reporting by participants. Although certain measures were taken during data collection to avoid systematic errors [33], there may still be information bias. Second, this study converts the HOMA-IR into a categorical variable for analysis, and also performs segmented stratified analysis. These operations may reduce the sample size in each group, leading to a decrease in test efficiency. Future studies could expand the sample size or focus on a specific subgroup for separate research.

## Conclusion

This study found that HOMA-IR was a reliable predictor of all-cause mortality in patients with CHD and hypertension. The relationship between HOMA-IR and all-cause mortality was U-shaped in this population. Both high or low HOMA-IR were associated with an increase in all-cause mortality.

## Data Availability

Data can be found at https://www.cdc.gov/nchs/nhanes/.

## Abbreviations

IR: Insulin resistance
CHD: Coronary heart disease
NHANES: National Health and Nutrition Examination Survey
NDI: National Death Index
NCHS: National Center for Health Statistics
HOMA-IR: Homeostatic model assessment of IR
TyG index: Triglyceride glucose index
TyG-BMI index: Triglyceride glucose-body mass index
ROC: Receiver operating characteristic
AUC: Area under the curve
PIR: Income -poverty ratio
COPD: Chronic obstructive pulmonary disease
LDL-C: Low density lipoprotein cholesterol
HDL-C: High density lipoprotein cholesterol
TC: Total cholesterol
ALT: Alanine aminotransferase
ALP: Alkaline phosphatase
AST: Aspartate aminotransferase
CK: Creatine kinase
Cr: Creatinine
GGT: Gamma-glutamyltransferase
LDH: Lactate dehydrogenase
HbA1c: Glycosylated hemoglobin
Hb: Hemoglobin
WBC: White blood cell count
